# Riemannian Means on Special Euclidean Group and Unipotent Matrices Group

**DOI:** 10.1155/2013/292787

**Published:** 2013-10-24

**Authors:** Xiaomin Duan, Huafei Sun, Linyu Peng

**Affiliations:** ^1^School of Mathematics, Beijing Institute of Technology, Beijing 100081, China; ^2^Department of Mathematics, University of Surrey, Guildford, Surrey GU2 7XH, UK

## Abstract

Among the noncompact matrix Lie groups, the special Euclidean group and the unipotent matrix group play important roles in both theoretic and applied studies. The Riemannian means of a finite set of the given points on the two matrix groups are investigated, respectively. Based on the left invariant metric on the matrix Lie groups, the geodesic between any two points is gotten. And the sum of the geodesic distances is taken as the cost function, whose minimizer is the Riemannian mean. Moreover, a Riemannian gradient algorithm for computing the Riemannian mean on the special Euclidean group and an iterative formula for that on the unipotent matrix group are proposed, respectively. Finally, several numerical simulations in the 3-dimensional case are given to illustrate our results.

## 1. Introduction

A matrix Lie group, which is also a differentiable manifold simultaneously, attracts more and more researchers' attention from both theoretic interest and its applications [1–5]. The Riemannian mean on the matrix Lie groups is widely studied for its varied applications in biomedicine, signal processing, and robotics control [6–9]. Fiori and Tanaka [10] suggested a general-purpose algorithm to compute the average element of a finite set of matrices belonging to any matrix Lie group. In [11], the author investigated the Riemannian mean on the compact Lie groups and proposed a globally convergent Riemannian gradient descent algorithm. Different invariant notions of mean and average rotations on SO(3) (it is compact) are given in [9]. Recently, Fiori [12] dealt with computing averages over the group of real symplectic matrices, which found applications in diverse areas such as optics and particle physics.

However, the Riemannian mean on the special Euclidean group SE(*n*) and the unipotent matrix group UP(*n*), which are the noncompact matrix Lie groups, has not been well studied. Fletcher et al. [6] proposed an iterative algorithm to obtain the approximate solution of the Riemannian mean on SE(3) by use of the Baker-Cambell-Hausdorff formula. In [7], the exponential mapping from the arithmetic mean of points on the Lie algebra *𝔰𝔢*(3) to the Lie group SE(3) was constructed to give the Riemannian mean in order to get a mean filter.

In this paper, the Riemannian means on SE(*n*) and those on UP(*n*), which are both important noncompact matrix Lie groups [13, 14], are considered, respectively. Especially, SE(3) is the spacial rigid body motion, and UP(3) is the 3-dimensional Heisenberg group *H*(3). Based on the left invariant metric on the matrix Lie groups, we get the geodesic distance between any two points and take their sum as a cost function. And the Riemannian mean will minimize it. Furthermore, the Riemannian mean on SE(*n*) is gotten using the Riemannian gradient algorithm, rather than the approximate mean. An iterative formula for computing the Riemannian mean on UP(*n*) is proposed according to the Jacobi field. Finally, we give some numerical simulations on SE(3) and those on *H*(3) to illustrate our results.

## 2. Overview of Matrix Lie Groups

In this section, we briefly introduce the Riemannian framework of the matrix Lie groups [15, 16], which forms the foundation of our study of the Riemannian mean on them.

### 2.1. The Riemannian Structures of Matrix Lie Groups

A group *G* is called a Lie group if it has differentiable structure: the group operators, that is, *G* × *G* → *G*, (*x*, *y*) ↦ *x* · *y* and *G* → *G*, *x* ↦ *x* ^−1^, are differentiable, *x*, *y* ∈ *G*. A matrix Lie group is a Lie group with all elements matrices. The tangent space of *G* at identity is the Lie algebra *𝔤*, where the Lie bracket is defined.

The exponential map, denoted by exp⁡, is a map from the Lie algebra *𝔤* to the group *G*. Generally, the exponential map is neither surjective nor injective. Nevertheless, it is a diffeomorphism between a neighborhood of the identity *I* on *G* and a neighborhood of the identity 0 on *𝔤*. The (local) inverse of the exponential map is the logarithmic map, denoted by log⁡.

The most general matrix Lie group is the general linear group GL(*n*, ℝ) consisting of the invertible *n* × *n* matrices with real entries. As the inverse image of ℝ − {0} under the continuous map *A* ↦ det⁡(*A*), GL(*n*, ℝ) is an open subset of the set of *n* × *n* real matrices, denoted by *M*_*n*×*n*_, which is isomorphic to ℝ^*n*×*n*^, it has a differentiable manifold structure (submanifold). The group multiplication of GL(*n*, ℝ) is the usual matrix multiplication, the inverse map takes a matrix *A* on GL(*n*, ℝ) to its inverse *A* ^−1^, and the identity element is the identity matrix *I*. The Lie algebra *𝔤𝔩*(*n*, ℝ) of GL(*n*, ℝ) turns out to be *M*_*n*×*n*_ with the Lie bracket defined by the matrix commutator

(1) $$\begin{array}{c} \left[X,Y\right]=XY-YX,\;\forall X,Y\in 𝔤𝔩\left(n,\mathbb{R}\right). \end{array}$$

All other real matrix Lie groups are subgroups of GL(*n*, ℝ), and their group operators are subgroup restrictions of the ones on GL(*n*, ℝ). The Lie bracket on their Lie algebras is still the matrix commutator.

Let *S* denote a matrix Lie group and *𝔰* its Lie algebra. The exponential map for *S* turns out to be just the matrix exponential; that is, given an element *X* ∈ *𝔰*, the exponential map is

(2) $$\begin{array}{c} \exp\left(X\right)=\overset{\infty }{\underset{m=0}{\sum }}\frac{X^{m}}{m!}. \end{array}$$

The inverse map, that is, the logarithmic map, is defined as follows:

(3) $$\begin{array}{c} \log\left(A\right)=\overset{\infty }{\underset{m=1}{\sum }}{\left(-1\right)}^{m+1}\frac{{\left(A-I\right)}^{m}}{m}, \end{array}$$

for *A* in a neighborhood of the identity *I* of *S*. The exponential of a matrix plays a crucial role in the theory of the Lie groups, which can be used to obtain the Lie algebra of a matrix Lie group, and it transfers information from the Lie algebra to the Lie group.

The matrix Lie group also has the structure of a Riemannian manifold. For any *A*, *B* ∈ *S* and *X* ∈ *T*_*A*_ *S*, the tangent space of *S* at *A*, we have the maps that

(4) LAB=AB,  (LA)∗X=AX,RAB=BA−1,  (RA−1)∗X=XA,

where *L* denotes the left translation, *R* denotes the right translation, and (*L*_*A*_)_∗_ and (*R*_*A*^−1^_)_∗_ are the tangent mappings associated with *L*_*A*_ and *R*_*A*^−1^_, respectively. The adjoint action Ad_*A*_ : *𝔰* → *𝔰* is

(5) $$\begin{array}{c} \text{A}{\text{d}}_{A}X=AXA^{-1}. \end{array}$$

It is also easy to see the formula that

(6) $$\begin{array}{c} \text{A}{\text{d}}_{A}=L_{A}R_{A}. \end{array}$$

Then, the left invariant metric on *S* is given by

(7) 〈X,Y〉A=〈(LA−1)∗X,(LA−1)∗Y〉I=〈A−1X,A−1Y〉I:=tr⁡((A−1X)TA−1Y)

with *X*, *Y* ∈ *T*_*A*_ *S* and tr denoting the trace of the matrix. Similarly, we can define the right invariant metric on *S* as well. It has been shown that there exist the left invariant metrics on all matrix Lie groups.

### 2.2. Compact Matrix Lie Group

A Lie group is compact if its differential structure is compact. The unitary group *U*(*n*), the special unitary group SU(*n*), the orthogonal group *O*(*n*), the special orthogonal group SO(*n*), and the symplectic group Sp(*n*) are the examples of the compact matrix Lie groups [17]. Denote a compact Lie group by *S*_1_ and its Lie algebra by *𝔰*_1_. There exists an adjoint invariant metric 〈·, ·〉 on *S*_1_ such that

(8) $$\begin{array}{c} \langle \text{A}{\text{d}}_{A}X,\text{A}{\text{d}}_{A}Y\rangle =\langle X,Y\rangle \end{array}$$

with *X*, *Y* ∈ *𝔰*_1_. Notice the fact that the left invariant metric of any adjoint invariant metric is also right invariant; namely, it is a bi-invariant metric; so all compact Lie groups have bi-invariant metrics. Furthermore, if the left invariant and the adjoint invariant metrics on *S*_1_ deduce a Riemannian connection ∇, then the following properties are valid:

(9) ∇XY=12[X,Y],〈ℛ(X,Y)X,Y〉=−14〈[X,Y],[X,Y]〉,

where *ℛ*(*X*, *Y*) is a curvature operator about the smooth tangent vector field on the Riemannian manifold (*S*_1_, ∇). Therefore, the section curvature *𝒦* is given by

(10) $$\begin{array}{c} 𝒦\left(X,Y\right)=\frac{\langle \left[X,Y\right],\left[X,Y\right]\rangle }{4\left(\langle X,X\rangle \langle Y,Y\rangle -{\langle X,Y\rangle }^{2}\right)}\geq 0, \end{array}$$

which means that *𝒦* is nonnegative on the compact Lie group.

In addition, according to the Hopf-Rinow theorem, a compact connected Lie group is geodesically complete. It means that, for any given two points, there exists a geodesic curve connecting them and the geodesic curve can extend infinitely.

### 2.3. The Riemannian Mean on Matrix Lie Group

Let *γ* : [0,1] → *S* be a sufficiently smooth curve on *S*. We define the length of *γ*(*t*) by

(11) ℓ(γ):=∫01〈γ˙(t),γ˙(t)〉γ(t)dt=∫01tr⁡{(γ(t)−1γ˙(t))Tγ(t)−1γ˙(t)}dt,

where *T* denotes the transpose of the matrix. The geodesic distance between two matrices *A* and *B* on *S* considered as a differentiable manifold is the infimum of the lengths of the curves connecting them; that is,

(12) d(A,B)≔inf⁡{ℓ(γ) ∣ γ:[0,1]→S with γ(0)=A,γ(1)=B}.

According to the Euclidean analogue (mean on Euclidean space), a definition of the mean of *N* matrices *R*_1_,…, *R*_*N*_ is the minimizer of the sum of the squared distances from any matrix to the given matrices *R*_1_,…, *R*_*N*_ on *S*. Now, we define the Riemannian mean based on the geodesic distance (12).

**Definition 1**

The mean of *N* given matrices *R*_1_,…, *R*_*N*_ on *S* in the Riemannian sense corresponding to the metric (7) is defined as

(13) $$\begin{array}{c} \bar{R}=\arg\underset{R\in S}{\min}\frac{1}{2N}\overset{N}{\underset{k=1}{\sum }}d{\left(R_{k},R\right)}^{2}. \end{array}$$

## 3. The Riemannian Mean on SE(*n*)

In this section, we discuss the Riemannian mean on the special Euclidean group SE(*n*), which is a subgroup of GL(*n* + 1, ℝ). Moreover, the special rigid body motion group SE(3) is taken as an illustrating example.

### 3.1. About SE(*n*)

The special Euclidean group SE(*n*) in ℝ^*n*^ is the semidirect product of the special orthogonal group SO(*n*) with ℝ^*n*^ itself [18]; that is,

(14) $$\begin{array}{c} \text{SE}\left(n\right)=\text{SO}\left(n\right)⋉{\mathbb{R}}^{n}. \end{array}$$

The matrix representation of elements of SE(*n*) is

(15) $$\begin{array}{c} \text{SE}\left(n\right)=\left\{\left(\begin{array}{cc} A & b\\ 0 & 1 \end{array}\right)\;|\;A\in \text{SO}\left(n\right),b\in {\mathbb{R}}^{n}\right\}. \end{array}$$

An element of SE(*n*) physically represents a displacement, where *A* corresponds to the orientation, or attitude, of the rigid body and *b* encodes the translation. The Lie algebra *𝔰𝔢*(*n*) of SE(*n*) can be denoted by

(16) $$\begin{array}{c} 𝔰𝔢\left(n\right)=\left\{\left(\begin{array}{cc} \Omega & v\\ 0 & 0 \end{array}\right)\;|\;\Omega^{T}=-\Omega ,\;v\in {\mathbb{R}}^{n}\right\}. \end{array}$$

Specially, when *n* = 3, the skew-symmetric matrix *Ω* can be uniquely expressed as

(17) Ω=(0−ωzωyωz0−ωx−ωyωx0)

with *ω* = (*ω*_*x*_, *ω*_*y*_, *ω*_*z*_) ∈ ℝ^3^. ||*ω*||_*F*_ gives the amount of rotation with respect to the unit vector along *ω*, where ||·||_*F*_ denotes the Frobenius norm. Physically, *ω* represents the angular velocity of the rigid body, whereas *v* corresponds to the linear velocity [19]. In [18], the author presents a closed-form expression of the exponential map *𝔰𝔢*(3) → SE(3) by

(18) $$\begin{array}{c} \exp\left(V\right)=I_{4}+V+\frac{1-\cos\left(\theta \right)}{\theta^{2}}V^{2}+\frac{\theta -\sin\left(\theta \right)}{\theta^{3}}V^{3} \end{array}$$

with *V* ∈ *𝔰𝔢*(3) and *θ* ^2^ = *ω*_*x*_^2^ + *ω*_*y*_^2^ + *ω*_*z*_^2^. Note that it can be regarded as an extension of the well-known Rodrigues formula on SO(3). The logarithmic map SE(3) → *𝔰𝔢*(3) is yielded as

(19) $$\begin{array}{c} \log\left(Q\right)=q_{1}\left(q_{2}I_{4}-q_{3}Q+q_{4}Q^{2}-q_{5}Q^{3}\right), \end{array}$$

where

(20) q1=18csc3(θ2)sec(θ2),q2=θcos⁡(2θ)−sin(θ),q3=θcos⁡(θ)+2θcos⁡(2θ)−sin(θ)−sin(2θ),q4=2θcos⁡(θ)+θcos⁡(2θ)−sin(θ)−sin(2θ),q5=θcos⁡(θ)−sin(θ),

tr⁡(*Q*) = 2 + 2cos⁡(*θ*), for −*π* < *θ* < *π*.

### 3.2. Algorithm for the Riemannian Mean on SE(*n*)

Denote *P*, *Q* ∈ SE(*n*) by

(21) $$\begin{array}{c} P=\left(\begin{array}{cc} A_{1} & b_{1}\\ 0 & 1 \end{array}\right),\;\;Q=\left(\begin{array}{cc} A_{2} & b_{2}\\ 0 & 1 \end{array}\right). \end{array}$$

Taking the corresponding exponential mappings on manifolds SO(*n*) and ℝ^*n*^ into consideration, the geodesic *γ*_*P*,*Q*_ between *P* and *Q* on the Lie group SE(*n*) is given by

(22) γP,Q(t)=(αA1,A2(t)βb1,b2(t)01)=(A1(A1TA2)tb1+(b2−b1)t01),

where *α* : [0,1] → SO(*n*) and *β* : [0,1] → ℝ^*n*^ are the geodesics expressed, respectively, by

(23) αA,B(t)=exp⁡A(tlog⁡(ATB))=A(ATB)t, A,B∈SO(n),βb1,b2(t)=exp⁡b1(t(b2−b1))=b1+(b2−b1)t, b1,b2∈ℝn.

Then, the midpoint of *P* and *Q* is defined by

(24) $$\begin{array}{c} P\circ Q=\left(\begin{array}{cc} A_{1}{\left(A_{1}^{T}A_{2}\right)}^{1/2} & \frac{1}{2}\left(b_{1}+b_{1}\right)\\ 0 & 1 \end{array}\right). \end{array}$$

Before the geodesic distance on SE(*n*) is given, we first introduce a lemma which is a known conclusion in linear algebra [20].

**Lemma 2**

If *E* ∈ ℝ^*n*×*n*^  and  *H* ∈ ℝ^*m*×*m*^ are invertible matrices, then the block matrix

(25) $$\begin{array}{c} \left(\begin{array}{cc} E & F\\ 0 & H \end{array}\right) \end{array}$$

is invertible, where *F* ∈ ℝ^*n*×*m*^. Furthermore,

(26) $$\begin{array}{c} {\left(\begin{array}{cc} E & F\\ 0 & H \end{array}\right)}^{-1}=\left(\begin{array}{cc} E^{-1} & -E^{-1}FH^{-1}\\ 0 & H^{-1} \end{array}\right). \end{array}$$

Now, we give the geodesic distance on SE(*n*) as follows.

**Lemma 3**

The geodesic distance between two points *P* and *Q* on SE(*n*) induced by the scale-dependent left invariant metric (7) is given by

(27) $$\begin{array}{c} d\left(P,Q\right)={\left({||\log\left(A_{1}^{T}A_{2}\right)||}_{F}^{2}+{||b_{2}-b_{1}||}_{F}^{2}\right)}^{1/2}. \end{array}$$

**Proof**

As mentioned above, the geodesic distance between two matrices *P* and *Q* on SE(*n*) is achieved by the length of geodesics connecting them; thus, we will compute it through substituting (22) into (11).

From Lemma 2, we get

(28) γP,Q−1(t) =((A1TA2)−tA1T−(A1TA2)−tA1T(b1+(b2−b1)t)01).

Then, according to the principle about the derivatives of the matrix-valued functions, the following formula is valid:

(29) γ˙P,Q(t)=(A1(A1TA2)tlog⁡(A1TA2)b2−b100).

Moreover, we have that

(30) tr⁡((γP,Q−1(t)γ˙P,Q(t))TγP,Q−1(t)γ˙P,Q(t))  =−log⁡2(A1TA2)+(b2−b1)T(b2−b1).

Therefore, the geodesic distance on SE(*n*) between *P* and *Q* is given by

(31) d(P,Q)=∫01(−log⁡2(A1TA2)+(b2−b1)2)1/2dt=(||log⁡(A1TA2)||F2+||b2−b1||F2)1/2.

This completes the proof of Lemma 2.

In addition, it is valuable to mention that the distance ||log⁡(*A*_1_^*T*^ *A*_2_)||_*F*_, induced by the standard bi-invariant metric on SO(*n*), stands for the rotation motion from the point *P* to *Q* and the distance ||*b*_2_ − *b*_1_||_*F*_ stands for the translation motion on ℝ^*n*^. Therefore, considering an object undergoing a rigid body Euclidean motion, then, this motion can be decomposed into a rotation with respect to the center of mass of the object and a translation of the center of mass of the object.

**Theorem 4**

For *N* given points on SE(*n*)

(32) Pk=(Akbk01),

where *A*_*k*_ ∈ SO(*n*)  and  *b*_*k*_ ∈ ℝ^*n*^, *k* = 1,2,…, *N*, if the Riemannian mean of *A*_1_, *A*_2_,…, *A*_*N*_ and the Riemannian mean of   *b*_1_, *b*_2_,…, *b*_*N*_ (i.e., arithmetic mean) are denoted by $\bar{A}$ and $\bar{b}$, respectively, then, one has the Riemannian mean $\bar{P}$ of *P*_1_, *P*_2_,…, *P*_*N*_ ∈ SE(*n*) by

(33) P¯=(A¯b¯01).

**Proof**

In the Riemannian sense, by (13), the mean $\bar{P}$ is defined as

(34) P¯=arg min⁡P∈SE(n)12N∑k=1Nd(Pk,P)2=arg min⁡P∈SE(n)12N∑k=1N(||log⁡(AkTA)||F2+||bk−b||F2)=arg min⁡A∈SO(n)12N∑k=1N||log⁡(AkTA)||F2+argmin⁡b∈ℝn12N∑k=1N||bk−b||F2.

From [9], the geodesic distance between *A*_*k*_ and *A* on SO(*n*) is given by

(35) $$\begin{array}{c} d{\left(A_{k},A\right)}^{2}={||\log\left(A_{k}^{T}A\right)||}_{F}^{2}, \end{array}$$

so we have that

(36) $$\begin{array}{c} \arg\underset{A\in \text{SO}(n)}{\min}\frac{1}{2N}\overset{N}{\underset{k=1}{\sum }}{||\log\left(A_{k}^{T}A\right)||}_{F}^{2}=\arg\underset{A\in \text{SO}(n)}{\min}d{\left(A_{k},A\right)}^{2}=\bar{A}. \end{array}$$

On the other hand, for *b*_*k*_ ∈ ℝ^*n*^, *k* = 1,2,…, *N*, it is easy to see that

(37) $$\begin{array}{c} \arg\underset{b\in {\mathbb{R}}^{n}}{\min}\frac{1}{2N}\overset{N}{\underset{k=1}{\sum }}{||b_{k}-b||}_{F}^{2}=\frac{1}{N}\overset{N}{\underset{k=1}{\sum }}b_{k}=\bar{b}. \end{array}$$

Therefore, the fact is shown that the Riemannian mean $\bar{b}$ of {*b*_*k*_} is equivalent to the arithmetic mean.

Consequently, we prove that equality (33) is valid.

In addition, let *L* denote the cost function of the minimization problem (34) on SE(*n*); that is,

(38) L(P)=Lrota(A)+Ltrans⁡(b)=12N∑k=1N||log⁡(AkTA)||F2+12N∑k=1N||bk−b||F2,

where *L*_rota_  and  *L*_trans⁡_ stand for the rotation and the translation components of the cost function *L*, respectively. We have the gradient of *L*_rota_(*A*) for *A* ∈ SO(*n*) as follows [21]:

(39) $$\begin{array}{c} \text{grad}\left(L_{\text{rota}}\right)=-A\overset{N}{\underset{k=1}{\sum }}\log\left(A^{T}A_{k}\right). \end{array}$$

Consequently, the Riemannian gradient descent algorithm is applied to calculate $\bar{A}$, taking the geodesic on SO(*n*) as the trajectory and the negative gradient (39) as the descent direction.

Finally, we achieve the following algorithm for computing the Riemannian mean $\bar{P}$ on SE(*n*).

**Algorithm 5**

Given *N* matrices *P*_*k*_, *k* = 1,2,…, *N*, on SE(*n*), their Riemannian mean $\bar{P}$ is computed by the following iterative method.

1. Store (1/*N*)∑_*k*=1_^*N*^ *b*_*k*_ to $\bar{b}$.
2. Set $\bar{A}=A_{1}$ as an initial input, and choose a desired tolerance *ε* > 0.
3. If ${||\sum_{k=1}^{N}\log({\bar{A}}^{T}A_{k})||}_{F}<\varepsilon$, then stop.
4. Otherwise, update $\bar{A}=\bar{A}\exp\left\{-\varepsilon \sum_{k=1}^{N}\log\left({\bar{A}}^{T}A_{k}\right)\right\}$, and go to step (3).

### 3.3. Simulations on SE(3)

**Figure 1:**
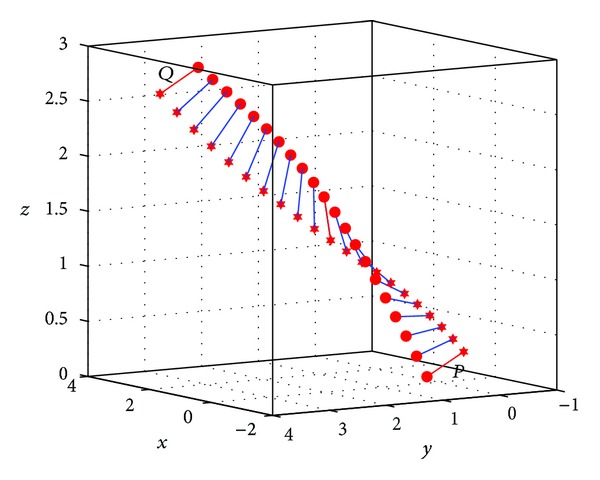
The rigid motion *D*(*t*) from *P* to *Q*.

**Figure 2:**
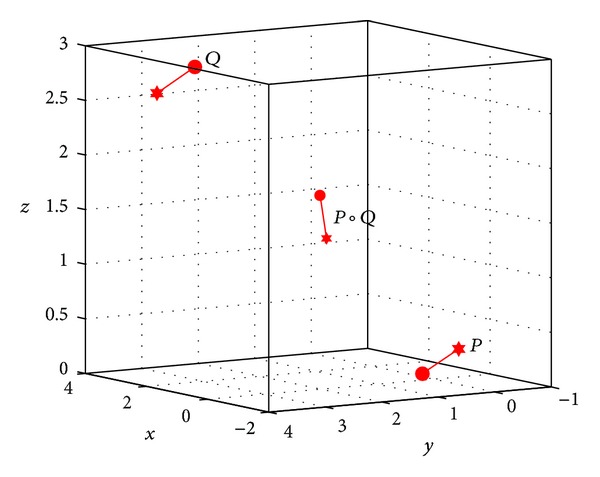
The Riemannian mean *P*∘*Q*.

Let us consider a rigid object *W* in the Euclidean space undergoing a rigid body Euclidean motion SE(3). Suppose that the coordinate of the center of gravity in *W* is *d*_*W*_ ∈ ℝ^3^; then, the optimal trajectory from the configuration *P* to *Q* is the curve *D*(*t*) such that

(40) $$\begin{array}{c} \left(\begin{array}{c} D\left(t\right)\\ 1 \end{array}\right)=\gamma_{P,Q}\left(t\right)\left(\begin{array}{c} d_{W}\\ 1 \end{array}\right), \end{array}$$

where *t* ∈ [0,1] and *γ*_*P*,*Q*_(*t*) denotes the geodesic connecting *P* and *Q* on SE(3)  (see Figure 1). For the configuration of two points *P* and *Q*, as shown in Figure 2, given by the angular velocity *ω*_*P*_,  *ω*_*Q*_ of the rigid body and the linear velocity *v*_*P*_, *v*_*Q*_, we choose *ω*_*P*_ = (*π*/2)(0,1, 1), *v*_*P*_ = (0,0, 0),  *ω*_*Q*_ = *π*(1/4,0, −1/2), and *v*_*Q*_ = (4.380, −1.348,3.690); then, we obtain their Riemannian mean according to Algorithm 5, which is just the middle point *P*∘*Q* from (24).

## 4. The Riemannian Mean on UP(*n*)

In this section, the Riemannian mean of *N* given points on the unipotent matrix group UP(*n*) is considered. UP(*n*) is a noncompact matrix Lie group as well. Moreover, in the special case *n* = 3, it is the Heisenberg group *H*(3).

### 4.1. About UP(*n*)

The set of all of the uppertriangular *n* × *n* matrices with diagonal elements that are all one is called unipotent matrices group, denoted by UP(*n*).

In fact, given an invertible matrix *C* ∈ UP(*n*), there is a neighborhood *U* of *C* such that every matrix in *U* is also in UP(*n*), so UP(*n*) is an open subset of ℝ^*n*×*n*^. Furthermore, the matrix product *P* · *Q* is clearly a smooth function of the entries of *P* and *Q*, and *P* ^−1^ is a smooth function of the entries of *P*. Thus, UP(*n*) is a Lie group. On the other hand, it can be verified that UP(*n*) is of dimension *n*(*n* − 1)/2 and is nilpotent. Since we can use the nonzero elements *C*_*ij*_,  *i* < *j*, directly as global coordinate functions for UP(*n*), the manifold underlying UP(*n*) is diffeomorphic to ℝ^*n*(*n*−1)/2^. Therefore, UP(*n*) is not compact, but simply connected.

The Lie algebra *𝔲𝔭*(*n*) of UP(*n*) consists of uppertriangular matrices *T* with diagonal elements *T*_*ii*_ = 0, *i* = 1,…, *n*. It is an indispensable tool which gives a realization of the Heisenberg commutation relations of quantum mechanics in the 3-dimensional case [17].

Moreover, it is the fact that both *C* − *I* and *T* are all nilpotent matrices, for any *C* ∈ UP(*n*) and *T* ∈ *𝔲𝔭*(*n*). Thus, from (2) and (3), the infinite series representations of the exponential mapping in *𝔲𝔭*(*n*) and the logarithm mapping in UP(*n*) can be given, respectively, by

(41) $$\begin{array}{c} \log\left(C\right)=\overset{n}{\underset{m=1}{\sum }}{\left(-1\right)}^{m+1}\frac{{\left(C-I\right)}^{m}}{m}, \end{array}$$

where *C* ∈ UP(*n*),  ||*C*−*I*||_*F*_ < 1, and

(42) $$\begin{array}{c} \exp\left(T\right)=\overset{n}{\underset{m=0}{\sum }}\frac{T^{m}}{m!} \end{array}$$

with *T* ∈ *𝔲𝔭*(*n*).

Notice that UP(*n*) is connected, which means that, for any given pair *A*, *B*, we can find a geodesic curve *γ*(*t*) such that *γ*(0) = *A* and *γ*(1) = *B*, namely, by taking the initial velocity as $\dot{\gamma }(0)=\log(A^{-1}B)$. Let the geodesic curve *γ*(*t*) be

(43) $$\begin{array}{c} \gamma \left(t\right)=A\exp\left(t\log\left(A^{-1}B\right)\right)\in \text{UP}\left(n\right) \end{array}$$

with *γ*(0) = *A*, *γ*(1) = *B*, and *γ*′(0) = log⁡(*A* ^−1^ *B*) ∈ *𝔲𝔭*(*n*). Then, the midpoint of *A* and *B* is given by

(44) $$\begin{array}{c} A\circ B=A\exp\left(\frac{1}{2}\log\left(A^{-1}B\right)\right), \end{array}$$

and from (11) the geodesic distance *d*(*A*, *B*) can be computed explicitly by

(45) $$\begin{array}{c} d\left(A,B\right)={||\log\left(A^{-1}B\right)||}_{F}. \end{array}$$

### 4.2. Algorithm for the Riemannian Mean on UP(*n*)

For *N* given points *B* ^1^, *B* ^2^,…, *B* ^*N*^ in UP(*n*), *L* denotes the cost function of the minimization problem (13); that is,

(46) $$\begin{array}{c} \underset{A\in \text{UP}\left(n\right)}{\min}L\left(A\right)=\underset{A\in \text{UP}(n)}{\min}\frac{1}{2N}\overset{N}{\underset{k=1}{\sum }}d{\left(B^{k},A\right)}^{2}. \end{array}$$

Following [22, 23], it has been shown that the Jacobi field is equal to zero at the Riemannian mean. The Jacobi field for the Riemannian mean is equal to the sum of tangent vectors to all geodesics (from mean to each point). Noticing the fact that the geodesic between two points *A* and *B* has already been given by (43), we can then compute the Jacobi field at point *A* to *N* points *B* ^*k*^ (at *t* = 0) such that

(47) γk(t)=A(A−1Bk)t=Aexp⁡(tlog⁡(A−1Bk)),dγk(t)dt|t=0=Alog⁡(A−1Bk), k=1,…,N.

Then, we suppose that the summation of all these vectors should be equal to zero; that is,

(48) $$\begin{array}{c} L_{A}=\overset{N}{\underset{k=1}{\sum }}{\frac{d\gamma_{k}\left(t\right)}{dt}|}_{t=0}=A\overset{N}{\underset{k=1}{\sum }}\log\left(A^{-1}B^{k}\right)=0, \end{array}$$

so the Riemannian mean *A* of the *N* matrices {*B* ^*k*^} should satisfy

(49) $$\begin{array}{c} \overset{N}{\underset{k=1}{\sum }}\log\left(A^{-1}B^{k}\right)=0. \end{array}$$

From the logarithm of the matrices on UP(*n*) given by (41), we can rewrite (49) as

(50) $$\begin{array}{c} \overset{N}{\underset{k=1}{\sum }}\overset{n}{\underset{m=1}{\sum }}{\left(-1\right)}^{m+1}\frac{{\left(A^{-1}B^{k}-I\right)}^{m}}{m}=0. \end{array}$$

Therefore, the Riemannian mean *A* of the *N* given matrices {*B* ^*k*^} can be given explicitly by solving (50).

For the case of *n* = 2, from (50), it is shown that the Riemannian mean ${\bar{A}}_{2}$ of *N* given matrices {*B*_2_^*k*^} in UP(2) is their arithmetic mean; that is,

(51) $$\begin{array}{c} {\bar{A}}_{2}=\frac{1}{N}\overset{N}{\underset{k=1}{\sum }}B_{2}^{k}. \end{array}$$

Next, for *n* = 3, we obtain the Riemannian mean on UP(3) (*H*(3)) as follows.

**Theorem 6**

Given *N* matrices {*B*_3_^*k*^} on the Heisenberg group *H*(3) by

(52) B3k=(1b12kb13k01b23k001),

where *k* = 1,2,…, *N*, then, one has the Riemannian mean ${\bar{A}}_{3}$ on the Heisenberg group *H*(3)  such that

(53) A¯3=(1b¯12b¯13−12cov⁡(b12,b23)01b¯23001),

where ${\bar{b}}_{ij}:=(1/N)\sum_{k=1}^{N}b_{ij}^{k}$,  *i*, *j* = 1,2, 3 (*i* < *j*), and $\mathrm{cov}(b_{12},b_{23}):=(1/N)\sum_{k=1}^{N}({\bar{b}}_{12}-b_{12}^{k})({\bar{b}}_{23}-b_{23}^{k})$.

**Proof**

First, let us denote the Riemannian mean ${\bar{A}}_{3}$ by

(54) A¯3=(1a12a1301a23001).

Then, note that, for the given matrices {*B*_3_^*k*^} on *H*(3), their Riemannian mean ${\bar{A}}_{3}$ has to satisfy (50), so we get the following solutions:

(55) a12=1N∑k=1Nb12k,a23=1N∑k=1Nb23k,a13=1N∑k=1N(a12−b12k)(a23−b23k).

As a matter of convenience, supposing that ${\bar{b}}_{ij}:=\left(1/N\right)\sum_{k=1}^{N}b_{ij}^{k}$,   *i*, *j* = 1,2, 3 (*i* < *j*), and $\mathrm{cov}(b_{12},b_{23}):=(1/N)\sum_{k=1}^{N}({\bar{b}}_{12}-b_{12}^{k})({\bar{b}}_{23}-b_{23}^{k})$, we show that (54) is valid.

This completes the proof of Theorem 6.

More generally, while *n* > 1, we can get the Riemannian mean on UP(*n*) given by the following theorem.

**Theorem 7**

Take *n* > 1. For *N* given matrices {*B*_*n*_^*k*^} in  UP(*n*), one assumes that they are in the form of

(56) Bnk=(Bn−1kbn−1k01)

with *B*_*n*−1_^*k*^ ∈ UP(*n* − 1) and *b*_*n*−1_^*k*^ ∈ ℝ^*n*−1^; then, the Riemannian mean ${\bar{A}}_{n}$ of the *N* matrices *B*_*n*_^*k*^ is given by

(57) A¯n=(A¯n−1an−101),

where ${\bar{A}}_{n-1}$ is the Riemannian mean of {*B*_*n*−1_^*k*^} and *a*_*n*−1_ is given by the formula that

(58) an−1=A¯n−1(∑k=1N∑m=0n−1(−1)mm+1(A¯n−1−1Bn−1k−I)m)−1×(∑k=1N∑m=0n−1(−1)mm+1(A¯n−1−1Bn−1k−I)mA¯n−1−1bn−1k).

**Proof**

For simplicity of exposition, we suppose that the Riemannian mean ${\bar{A}}_{n}$ is the block matrix in the form of

(59) A¯n=(An−1an−101)

with *A*_*n*−1_ ∈ UP(*n* − 1) and *a*_*n*−1_^*k*^ ∈ ℝ^*n*−1^. Since the Riemannian mean ${\bar{A}}_{n}$ of the *N* matrices {*B*_*n*_^*k*^} should satisfy (50), we substitute the block matrix forms (59) and (57) into (50). Then, we obtain the following matrix equation for the Riemannian mean ${\bar{A}}_{n}$:

(60) ∑k=1N∑m=1n(−1)m−1m ×((An−1−1Bn−1k−I)m(An−1−1Bn−1k−I)m−1An−1−1(bn−1k−an−1)00)=0,

which means that (58) is valid and *A*_*n*−1_ satisfies the equation

(61) $$\begin{array}{c} \overset{N}{\underset{k=1}{\sum }}\overset{n}{\underset{m=1}{\sum }}\frac{{\left(-1\right)}^{m-1}}{m}{\left(A_{n-1}^{-1}B_{n-1}^{k}-I\right)}^{m}=0. \end{array}$$

Moreover, from (41), we have that

(62) $$\begin{array}{c} \log\left(A_{n-1}^{-1}B_{n-1}^{k}\right)=0. \end{array}$$

Furthermore, it is shown that *A*_*n*−1_ is the Riemannian mean of {*B*_*n*−1_^*k*^}. At last, we write *A*_*n*−1_ as ${\bar{A}}_{n-1}$, so the proof of Theorem 7 is completed.

As shown above, we give the iterative formula for computing the Riemannian mean for any dimension *n* > 1. Either (51) or (54) can be chosen as the initial formula.

### 4.3. Simulations on *H*(3)

In this section, we take two examples to illustrate the results about the Riemannian mean on the Heisenberg group *H*(3), which is the 3-dimensional space.

**Example 8**

**Figure 3:**
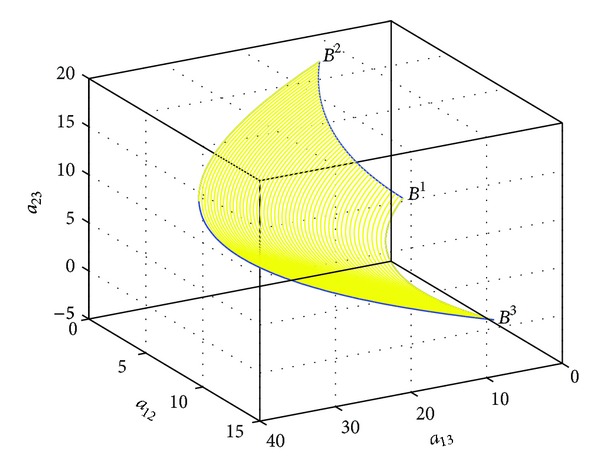
The geodesic triangle on *H*(3).

**Figure 4:**
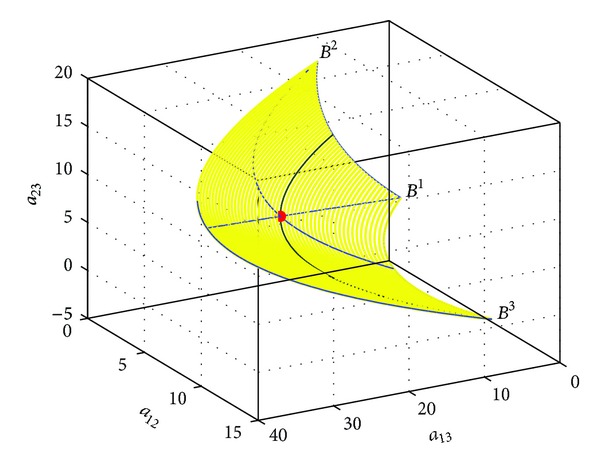
The Riemannian mean of three points.

Consider the Riemannian mean of three points *B* ^1^,  *B* ^2^,  *B* ^3^ on the Heisenberg group *H*(3). Using (43), we can get the geodesics of three points on *H*(3), which form a geodesic triangle. In Figure 3, all of the curves are geodesics. Moreover, as shown in Figure 4, the midpoint of each geodesic is easy to be obtained by (44). Thus, each centerline connects a vertex to the midpoint of its opposing side. On *H*(3), these centerlines always meet in a single point which is coincident with the Riemannian mean computed by (54), denoted by a red dot as shown in Figure 4.

**Example 9**

**Figure 5:**
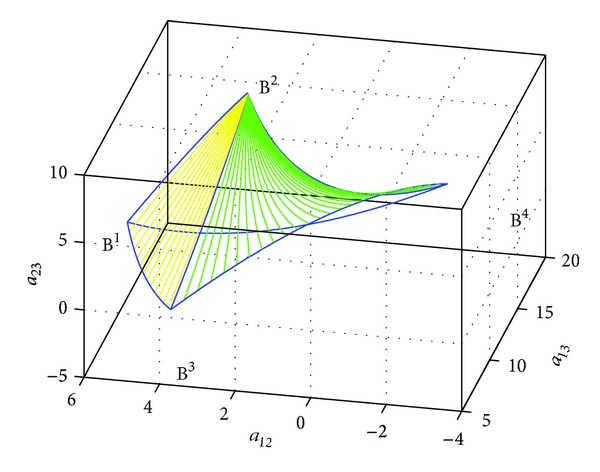
The geodesic tetrahedron on *H*(3).

**Figure 6:**
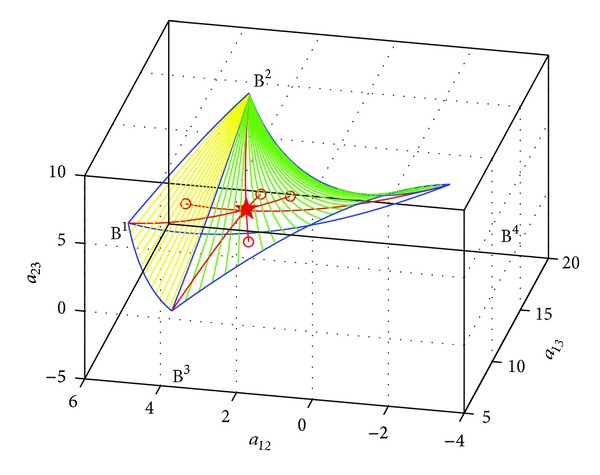
The Riemannian mean of four points.

Given four points *B* ^1^,  *B* ^2^,  *B* ^3^,  *B* ^4^ on the Heisenberg group *H*(3), we can get a geodesic tetrahedron from (43) (see Figure 5), where all curves are geodesics. Moreover, similar to Example 8, the Riemannian means of three vertexes on each curved face are obtained, denoted by red circles (see Figure 6). Then, we plot each centerline which connects a vertex to the Riemannian mean of its opposing side. It is shown that these centerlines still meet in a single point, denoted by a red pentacle. In fact, the point is the Riemannian mean of *B* ^1^,  *B* ^2^,  *B* ^3^,  *B* ^4^ applying (54).

## 5. Conclusion

In this paper, we consider the Riemannian means on the special Euclidean group SE(*n*) and the unipotent matrix group UP(*n*), respectively. Based on the left invariant metric on the matrix Lie groups, we get the geodesic distance between any two points and take their sum as a cost function. Furthermore, we get the Riemannian mean on SE(*n*) using the Riemannian gradient algorithm. Moreover, we give an iterative formula for computing the Riemannian mean on UP(*n*) according to its Jacobi field. Finally, we make advantages of several numerical simulations on SE(3) and *H*(3) to illustrate our results.
